# Impacts of ocean acidification on marine organisms: quantifying sensitivities and interaction with warming

**DOI:** 10.1111/gcb.12179

**Published:** 2013-04-03

**Authors:** Kristy J Kroeker, Rebecca L Kordas, Ryan Crim, Iris E Hendriks, Laura Ramajo, Gerald S Singh, Carlos M Duarte, Jean-Pierre Gattuso

**Affiliations:** * Bodega Bay Laboratory, University of California2099 Westside Rd, Bodega Bay, CA, 94923, USA; † University of British ColumbiaVancouver, BC, Canada, V6T1Z4; ‡ Puget Sound Restoration Fund590 Madison Ave N, Bainbridge Island, WA, 98110, USA; § Global Change department, IMEDEA (CSIC-UIB), Instituto Mediterráneo de Estudios AvanzadosC/Miquel Marqués 21, Esporles (Mallorca), 07190, Spain; ¶ Laboratorio de Ecologia y Cambio Climatico, Facultad de Ciencias Universidad Santo TomasC/Ejercito, 146, Santiago de Chile; ‖ The UWA Oceans Institute and School of Plant Biology, University of Western Australia35 Stirling Highway, Crawley, 6009, Australia; ** Laboratoire d'Océanographie de Villefranche-sur-Mer, CNRS-INSUBP 28, Villefranche-sur-Mer Cedex, 06234, France; †† Université Pierre et Marie Curie-Paris 6Observatoire Océanologique de Villefranche, Villefranche-sur-Mer Cedex, 06230, France

**Keywords:** calcification, carbonate chemistry, climate change, cumulative effects, pH

## Abstract

Ocean acidification represents a threat to marine species worldwide, and forecasting the ecological impacts of acidification is a high priority for science, management, and policy. As research on the topic expands at an exponential rate, a comprehensive understanding of the variability in organisms' responses and corresponding levels of certainty is necessary to forecast the ecological effects. Here, we perform the most comprehensive meta-analysis to date by synthesizing the results of 228 studies examining biological responses to ocean acidification. The results reveal decreased survival, calcification, growth, development and abundance in response to acidification when the broad range of marine organisms is pooled together. However, the magnitude of these responses varies among taxonomic groups, suggesting there is some predictable trait-based variation in sensitivity, despite the investigation of approximately 100 new species in recent research. The results also reveal an enhanced sensitivity of mollusk larvae, but suggest that an enhanced sensitivity of early life history stages is not universal across all taxonomic groups. In addition, the variability in species' responses is enhanced when they are exposed to acidification in multi-species assemblages, suggesting that it is important to consider indirect effects and exercise caution when forecasting abundance patterns from single-species laboratory experiments. Furthermore, the results suggest that other factors, such as nutritional status or source population, could cause substantial variation in organisms' responses. Last, the results highlight a trend towards enhanced sensitivity to acidification when taxa are concurrently exposed to elevated seawater temperature.

## Introduction

Ocean acidification is projected to impact all areas of the ocean, from the deep sea to coastal estuaries (Orr *et al*., 2005; Feely *et al*., 2009, 2010), with potentially wide-ranging impacts on marine life (Doney *et al*., 2009). There is an intense interest in understanding how the projected changes in carbonate chemistry will affect marine species, communities, and ecosystems (Logan, 2010; Gattuso & Hansson, 2011a). The rapidly growing body of experimental research on the biological impacts of acidification spans a broad diversity of marine organisms and reveals an even broader range of species' responses, from reduced calcification rates in oysters (e.g., Gazeau *et al*., 2007; Talmage & Gobler, 2010; Waldbusser *et al*., 2011) to impaired homing ability in reef fishes (Munday *et al*., 2009, 2010) to increased growth rates in macro algae (Hurd *et al*., 2009; Koch *et al*., 2013). Translating the wide range of responses to ecosystem consequences, management actions, and policy decisions requires a synthetic understanding of the sources of variability in species responses to acidification and the corresponding levels of certainty of the impacts.

Meta-analysis is a quantitative technique for summarizing the results of primary research studies. It provides a transparent method to identify key patterns across numerous studies, and can be used to develop hypotheses for future research. Furthermore, it can be a powerful tool for placing individual studies into the context of a broader field of research on a topic. While several meta-analyzes have been published regarding ocean acidification (Dupont *et al*., 2010; Hendriks *et al*., 2010; Kroeker *et al*., 2010; Liu *et al*., 2010), research on this topic is growing exponentially (Gattuso & Hansson, 2011b). Over 403 studies investigating ocean acidification have been published since the beginning of 2010, which more than triples the number of studies included in any previous meta-analysis of its impacts (Hendriks *et al*., 2010; Kroeker *et al*., 2010; Liu *et al*., 2010). These new studies provide an important opportunity to expand our understanding of species vulnerability and resilience to ocean acidification by including a broader array of species in the analyzes, as well as an opportunity to test the robustness of the patterns found in previous analyzes and highlight new insights.

Previous meta-analyzes identified significant variation in response to ocean acidification among broad taxonomic groups (Kroeker *et al*., 2010) and suggested there is predictable sensitivity among heavily calcified organisms and higher tolerance among more active mobile organisms (e.g., crustaceans and fish). Variation in sensitivity among calcifying taxa was primarily attributed to differences in life history characteristics, including the degree of control over calcification processes (Berry *et al*., 2002; Cohen *et al*. 2009), the presence or absence of biogenic coverings that separate calcified material from seawater (e.g., the periostracum in mussels Ries *et al*., 2009; Rodolfo-Metalpa *et al*., 2011), or the amount of calcium carbonate in an organism's shell or skeleton (Kroeker *et al*., 2011a). However, there is still unresolved variation in sensitivity *within* these taxonomic groups. Determining whether the remaining variation within taxonomic groups is due to species-specific differences that are inherently difficult to predict, or is due to additional methodological or biological factors remains an important area of research.

Several hypotheses regarding the variation in sensitivity to ocean acidification have been proposed that are not directly related to taxonomic characteristics. For example, acidification's effects can differ across life stages of the same species (e.g., Talmage & Gobler, 2010; Albright & Langdon, 2011; Crim *et al*., 2011; Martin *et al*., 2011). Pronounced sensitivity among a particular life history stage could determine the sensitivity of the species as a whole, but previous meta-analyzes were not able to detect clear patterns among life history stages when all taxa were pooled together (Kroeker *et al*., 2010). It was proposed that differences among life stages may be apparent within taxonomic groups, but the lack of studies at the early life history stages of many taxa prevented these comparisons (Kroeker *et al*., 2010). Therefore, the emergence of numerous studies on larvae in recent years, may allow a re-evaluation of acidification's impacts across different life history stages.

Recent research has highlighted other factors that may underlie variability in sensitivity among and within taxonomic groups. For example, increased food or nutrient supply can offset reductions in calcification and growth associated with acidification in corals (Cohen *et al*. 2009; Holcomb *et al*., 2010) and mussels (Melzner *et al*., 2011; Thomsen *et al*., 2013). Furthermore, adaptation can cause one population to be more or less sensitive than another population of the same species (Langer *et al*., 2009; Parker *et al*., 2011). In addition, some species may be able to acclimate to acidification over longer time frames (Form & Riebesell, 2011), suggesting that the duration of the experiment may influence the species response. As research on ocean acidification has progressed, it is important to understand how the variability due to these factors compares to other known sources of variation.

Moreover, the increasing levels of atmospheric CO_2_ are concurrently driving ocean warming (Meehl *et al*. 2007), and a growing number of experiments have tested the combined effect of ocean acidification and warming. Elevated temperatures can increase the metabolic rate of organisms within their thermal tolerance window, but cause a rapid deterioration of cellular processes and performance beyond tolerance limits (Pörtner, 2008). Hence, predicting the combined effects of warming and acidification is difficult, as warming could either offset the effects of ocean acidification (McCulloch *et al*., 2012) or aggravate it through an accumulation of stress effects (Anthony *et al*., 2008). As a result, meta-analyzes on the impacts of ocean acidification can now extend beyond preceding efforts by addressing the role of warming on the response of marine biota to acidification.

As research has progressed, it is important to examine how new studies influence our understanding of acidification's impacts. Here, we test the robustness of previous conclusions regarding the sensitivity of various taxonomic groups to ocean acidification to an additional 155 studies (representing approximately 100 new species that were not included in the previous meta-analysis (Kroeker *et al*., 2010), which had 79 species). In particular, we used meta-analyzes to test: (i) how taxa vary in key physiological responses, as well as changes in abundance to ocean acidification; (ii) how these effects vary across different life stages within common taxonomic groups; and (iii) how increased temperatures influence the effect of acidification across multiple response variables. We then compare these results to previous analyzes and highlight new insights.

## Materials and methods

For these analyzes, we repeated the methods reported in Kroeker *et al*. (2010). First, we identified studies that measured any biological response to ocean acidification published from 1 January 2010 to 1 January 2012 by searching ISI web of science and the European Project on Ocean Acidification (EPOCA) blog (http://oceanacidification.wordpress.com/), as well as the literature cited of the identified studies, resulting in 403 published studies.

We included the data from any study that measured a biological response to a 0.5 unit reduction or less in mean seawater pH (on any pH scale), which reduced the 403 studies to 155 studies. The 0.5 unit reduction in pH was chosen to approximate projections for changes in the global mean surface pH in the near future (i.e., 2100) (Caldeira & Wickett, 2003; Caldeira, 2005). Although the magnitude of projected pH reductions varies by location and depth (Feely *et al*., 2009), we chose the response to a pH change closest to this global projection (<0.5) to minimize experimental variation. However, we then tested the effect of the magnitude of pH changes on our response estimates (see sensitivity analyzes below).

Although multiple carbonate chemistry parameters will change with acidification, we chose to compare responses with mean reductions in pH, because it is the most commonly reported seawater chemistry parameter that allowed us to best standardize comparisons among experiments. In addition, we chose to use a relative change in pH from the control pH designated by the author of each study (rather than particular pH or pCO_2_ values) to allow for differences in the ambient (control) conditions in the system of interest. However, there are still many studies that do not adequately characterize the carbonate chemistry for their study system to know if the designated control is ecologically relevant, and instead rely on global mean pCO_2_ levels and projections, despite research that has highlighted the wide range of pH values marine organisms are currently experiencing (e.g., Hofmann *et al*., 2011). While this is an important area for improvement (McElhany & Busch, 2012), we rely on the authors' designations of control pH for the current analysis, which range from pH_T_ 7.8 to 8.2. The pH total scale is used throughout the study when absolute pH values are indicated.

Data from any experiment that factorially manipulated both carbonate chemistry and temperature were also collected. For these experiments, we analyzed responses at ambient and a 2–3 °C elevated temperature treatment to approximate the projected *global averages* of near-future warming in the surface ocean (IPCC, 2007). While warming is projected to be more extreme in some areas, all studies had similar temperature manipulations (2–3 °C), which allowed us to standardize among studies.

The choice of which studies to include in meta-analysis can profoundly influence the conclusions (Abrami *et al*., 1988; Englund *et al*., 1999; Osenberg *et al*., 1999). It is recommended that all relevant data are included in the meta-analysis and that decisions regarding whether studies should be included based on judgments of ‘quality’ be minimized due to issues of bias (Englund *et al*., 1999). Instead, running and reporting multiple meta-analyzes with various levels of data selection criteria is recommended to test the robustness of the patterns. Thus, all studies that measured a biological response to a 0.5 unit reduction in pH were included, and several analyzes were used to test the role of data selection criteria and potential methodological sources of variation (Osenberg *et al*., 1999). Data points and error estimates were obtained from the EPOCA database (Nisumaa *et al*., 2010) or interpolated from figures with graphical software (data thief iii v. 1.5, Amsterdam, the Netherlands; and graphclick v. 3.0, Neuchâtel, Switzerland).

The data set, comprised of 155 studies, which was then merged with another data set (built with the same methods) that was based on studies published prior to 1 January 2010 (Kroeker *et al*., 2010). This combined data set had 228 studies, measuring responses of marine organisms to ocean acidification (Table S1). For each study (i.e., a published article), responses from separate experiments (i.e., independent experiments within a published article) at ambient levels of any other factors (e.g., temperature, nutrients, food supply, light levels) were collected. When ambient food concentrations were not reported, we included the responses of the fed/higher nutrient treatments over the unfed/lower nutrient concentrations. In addition, the differences in responses between the fed/high nutrient and unfed/low nutrient responses were compared with the mean effects and variability for given responses.

Responses from separate species in the same experiment (e.g., species allowed to interact in the same tank) were collected separately. Although, the responses of multiple species from the same experiment are not truly independent, we chose to include multiple species responses from a single experiment, because the indirect effects (e.g., species interactions) of acidification that are nonindependent are very pertinent to global acidification scenarios where species will be experiencing both direct and indirect effects. In addition, multiple lines/populations of the same species from the same experiment were all included for similar reasons. Differences between lines/populations of the same species represent real sources of variability that are the focus of this study. The entire data set primarily consisted of experiments on single species, but also included field experiments (e.g., 18 studies from natural gradients and naturally acidified ecosystems and 21 studies using mesocosms with multiple species).

For each experiment, the effect of acidification was calculated as the log-transformed response ratio (*LnRR*). It is the ratio of the mean effect in the acidification treatment to the mean effect in a control group (Hedges *et al*., 1999). Then, the overall mean effect was calculated for each response variable (survival, calcification, growth, photosynthesis, development, abundance, and metabolism) by weighing each individual *LnRR* by the inverse of the sum of its sampling variance and the between experiment variance, and then calculating the weighted mean (i.e., random effects meta-analysis; Hedges & Olkin, 1985). Because of the weighting by variance, any experiment that did not report an error estimate was excluded from the random effects meta-analysis. This resulted in 29 responses excluded from the main analyzes (although they were included in a sensitivity analysis; Fig. S1). When a single experiment reported several response variables, we included only one response from an experiment per response variable to avoid pseudoreplication. For example, if an experiment reported the effects on calcification, growth rate, and metabolism, each of those responses were included in the separate meta-analyzes for each response. However, if an experiment reported the effects on various metrics of a response type, such as growth rates based on changes in biomass and length, we included only the most inclusive for that response variable (i.e., we chose to use biomass rather than length to represent growth).

Calcification responses were primarily the estimates of net calcification. Growth responses included estimates of change in biomass, length, width, somatic tissue, and growth rates. Photosynthesis responses included changes in the photosynthetic rate or efficiency. Development responses were primarily based on indices of embryonic or larval development (e.g., percent metamorphosed, percent larvae to reach a certain stage, etc.). Abundance responses encompassed the number of individuals, including the number of newly settled individuals, as well as percent cover estimates. Survival rates were typically reported as the final percent survival or mortality at the end of the experiment, which were then converted to survival. In addition to the analyzes on this raw data, the survival data were also converted into specific daily survival rates to account for differences in the duration of the experiments, and unweighted fixed effects meta-analyzes were performed on *LnRR* estimates on these duration-weighted, daily survival rates (Fig. S2). Because the focus of this study includes only key physiological and ecological parameters, it should be noted that there are likely to be important effects of ocean acidification that are not captured in this analysis. Several studies report the effects of ocean acidification on reproduction (e.g., fertilization success). However, because this is the subject of several qualitative reviews (Albright, 2011; Byrne, 2011; Ross *et al*., 2011), it is not considered here.

Heterogeneity in mean effect sizes was determined by a significant (α = 0.05) *Q*_T_ statistic, which is calculated by summing the standard deviation of each effect size from the overall mean effect size estimate, and then weighting each one by the inverse of its sampling variance (Cochran, 1954; Rosenberg *et al*., 2000). Significant heterogeneity (*Q*_T_) can indicate that there is underlying data structure that is not adequately captured by the mean effect size (e.g., multiple populations of effect sizes rather than just one population of effect sizes), potentially signaling important sources of biological variation.

The variation in effect sizes among (i) taxa; and (ii) life stages within taxonomic groups was tested with categorical random effects meta-analysis (Hedges & Olkin, 1985). For these analyzes, effect sizes were first partitioned into categories (based on taxonomic groups or life stages within taxonomic groups, respectively). Only the response variables with representative studies in the priori defined categories, and only those categories that had four or more data points for the analyzes were included. The statistic *Q*_M_ (which quantifies the variation explained by the chosen categories vs. the residual variation, which is defined by *Q*_E_) was then computed to determine whether significant variability is explained by the categories (Hedges & Olkin, 1985; Rosenberg *et al*., 2000). The significance of *Q*_M_ was tested by a randomization procedure that randomly re-assigns the effect sizes to the categories to create a probability distribution for mean effect sizes of each category using 9999 iterations (Rosenberg *et al*., 2000).

Variation in the effects of acidification at ambient and elevated seawater temperatures was tested by analyzing only those studies that factorially compared both factors (i.e., <0.5 unit reduction in pH combined with a 2–3 °C rise in seawater temperature). We only analyzed the effect of acidification at the ambient seawater temperature (identified by the author of the primary study) in the previous analyzes. In the present analysis, a random effects categorical meta-analysis was performed on (i) the effect of acidification at ambient temperature; (ii) the effect of acidification at an elevated temperature for each different response variable. All meta-analyzes were performed with metawin V. 2.0 (Sinauer Associates).

After meta-analyzes, the mean *LnRR* estimates were back transformed to mean percent change estimates for ease of interpretation. Because each response ratio was natural log-transformed prior to calculating the mean effect size, the antilog of the mean *LnRR* was taken to calculate a mean response ratio. Back transformations using the antilog provide a geometric mean of the response ratios, which is known to underestimate the arithmetic mean (Rothery, 1988). However, the underestimation of the arithmetic mean is generally very small (Hedges *et al*., 1999). Therefore, reported mean percent change transformations can be considered conservative estimates.

### Sensitivity analyzes

To examine the robustness of the results, the Rosenthal's fail-safe number was calculated for each analysis. It estimates the number of nonsignificant results needed to change the significance of the meta-analysis. Furthermore, the disproportionate contribution of an individual experiment with a large magnitude effect size to a given result was tested by (i) ranking each experiment by the magnitude of its effect size; and (ii) individually removing each of the five experiments with the largest magnitude effect sizes from the overall analyzes one at a time and re-running the analyzes. If the exclusion of a single experiment changed the significance of the overall mean effect size or the heterogeneity statistic (*Q*_T_), we would want to consider removing it from the analysis as it would signal a disproportionate contribution to the overall result. However, this was not the case in any analysis, and all experiments were included. Normality was also checked with normal quantile plots, and non-normal distributions were compensated for by testing the significance of *Q*_T_ and *Q*_M_ statistics with randomization tests from 9999 iterations of the data and bootstrapped bias-corrected 95% confidence intervals for the mean effect sizes (Adams *et al*., 1997).

Unweighted, fixed effects meta-analyzes were also run for each dataset to examine the role of data selection and weighting on the results (Englund *et al*., 1999). This allowed the inclusion of studies that did not report error estimates and that were excluded from the weighted analyzes. Finally, differences in effects sizes due to methodological factors, such as length of experiment or magnitude of pH change, were tested with continuous random-effects meta-analysis (Rosenberg *et al*., 2000). Separate analyzes were performed for each taxonomic group with more than 10 data points with either duration of experiment or magnitude of pH change as a continuous variable.

## Results

**Fig. 1:**
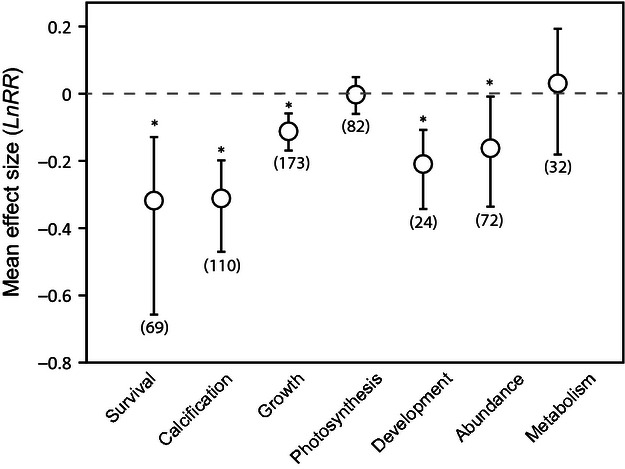
Mean effect of near future acidification on major response variables. Significance is determined when the 95% bootstrapped confidence interval does not cross zero. The number of experiments used to calculate the mean is included in parentheses. *denotes a significant effect.

When all taxa are pooled together, ocean acidification had a significant negative effect on survival, calcification, growth, development and abundance (Fig. 1; Table S2). Overall, survival and calcification are the responses most affected by acidification, with 27% reductions in both responses, whereas growth and development are reduced by approximately 11–19%, respectively, for conditions roughly representing year 2100 scenarios. On average, the abundance is reduced 15%. In contrast, effects of acidification on photosynthesis and metabolism are not detected, when all taxa are pooled together.

**Fig. 2:**
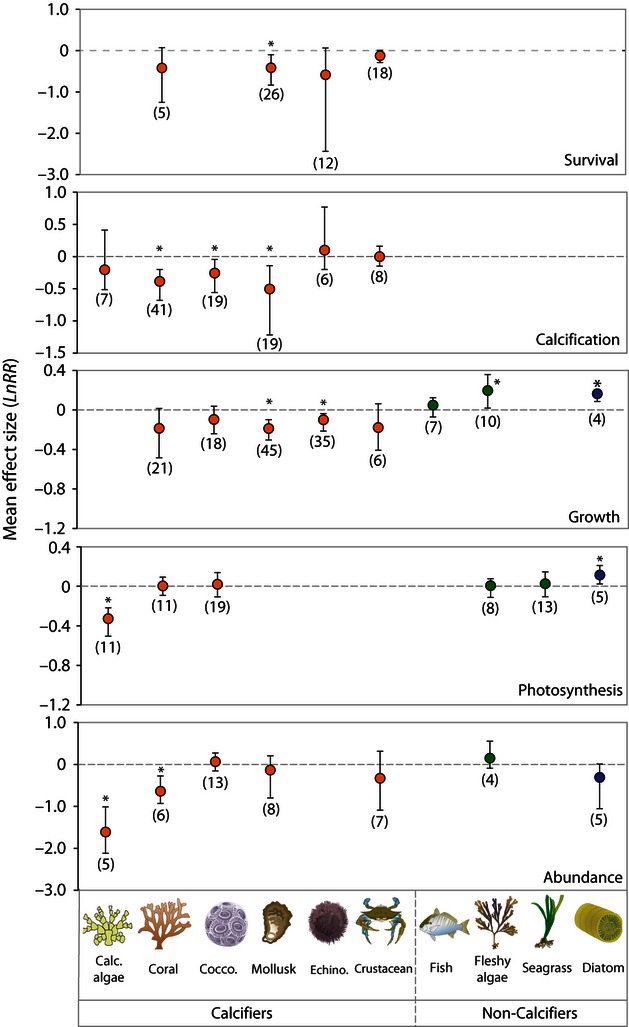
Variation in effect sizes among key taxonomic groups, divided by major response variables. Note there are different scales on the y-axes to highlight the variation among taxa. Means are from a weighted, random-effects model with bootstrapped bias-corrected 95% confidence intervals. The number of experiments used to calculate the means is given in parentheses. Not all response variables are considered in this analysis. *denotes a significant difference from zero.

**Fig. 3:**
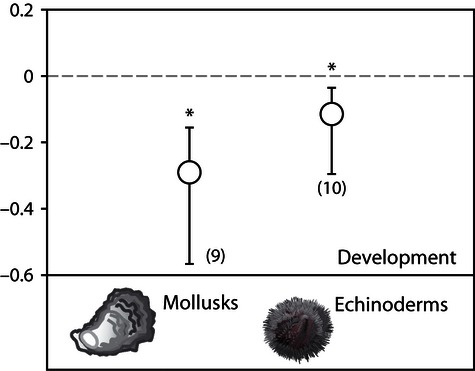
Variation in effects of acidification among taxa for development. Means are from weighted, random effects meta-analysis and are shown with bias-corrected bootstrapped 95% confidence intervals. The number of experiments used to calculate each mean is given in parentheses. *denotes a significant difference from zero.

**Fig. 4:**
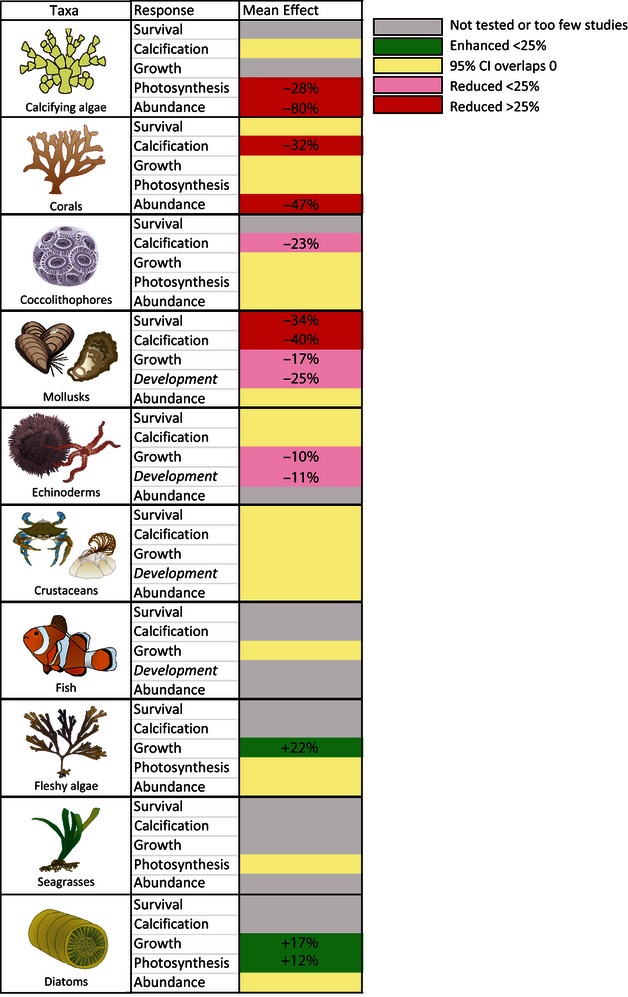
Summary of effects of acidification among key taxonomic groups. Effects are represented as either mean percent (+) increase or percent (−) decrease in a given response. Percent change estimates were back transformed from the mean *LnRR*, and represent geometric means, that are conservative of the arithmetic means.

The magnitude of these effects varies among taxa (Figs 2–4; Table S3). Reductions in survival are similar among corals, mollusks and echinoderms (although only significant for mollusks), whereas no effect is detected for crustaceans. Corals, coccolithophores, and mollusks show the greatest mean reductions in calcification (22–39%), whereas a significant mean effect of acidification is not detected on the calcification of echinoderms or crustaceans. However, these differences among taxonomic groups are not significant sources of variation in this analysis (Table S3). All calcified taxa show similar magnitude mean reductions in growth (9–17% reductions), although these reductions are only statistically significant for mollusks and echinoderms. Effects on fish growth are not detected, whereas growth increases 22% on average among fleshy algae and 18% among diatoms (growth *Q*_M__8,146_ = 70.85, *P* = 0.001). The effects of acidification on photosynthesis vary little among taxa with the exception of calcified algae, for which photosynthesis is reduced 28% on average (photosynthesis *Q*_M 5,61_ = 40.88, *P* = 0.004). This sensitivity in calcified algae is also apparent in experiments that tested for impacts on abundance, where calcified algae have a much greater mean reduction (80%) in percent cover/abundance in acidified conditions than other groups. In addition, corals suffer significant mean reductions in abundance (47%) in acidified treatments, whereas there is very high variability among other taxa (abundance *Q*_M__6,41_ = 42.55, *P* = 0.005).

**Fig. 5:**
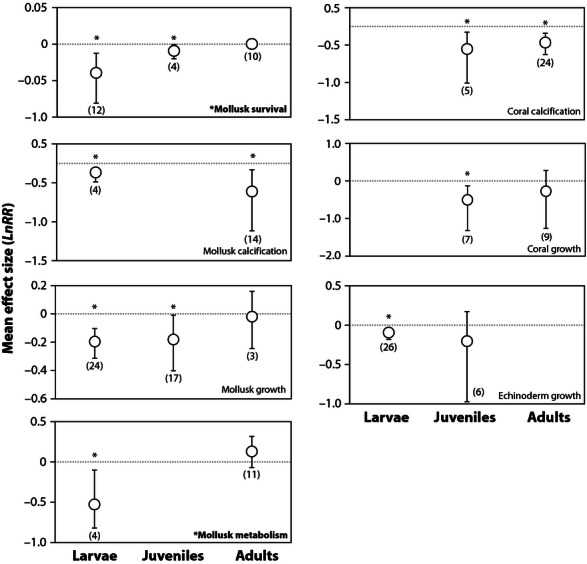
Significant variation in the effects of near-future ocean acidification among lifestages within taxonomic groups. Error bars represent bias-corrected bootstrapped 95% confidence intervals, and the number of experiments used to calculate the means is shown in parentheses. The * associated with mollusk survival and metabolism denotes a significant difference in effect size among life history stages (Significant *Q*_M_).

In addition, acidification reduces the development of the early life stages of mollusks and sea urchins (Fig. 3; bivalves dominate the mollusk category in 9 of 13 experiments). In comparisons among life stages, the mean effect of acidification on mollusk survival was lowest for larvae (*Q*_M__2,23_ = 3.22, *P* = 0.05; Fig. 5; Table S4). This pattern is consistent for the effects of acidification on mollusk metabolism (primarily estimated by oxygen consumption); metabolism is significantly reduced among mollusk larvae and unaffected or increased slightly among adults (*Q*_M__1,13_ = 15.82, *P* = 0.003; Fig. 5). No significant differences in effect sizes are detected among life stages within taxonomic groups for any other response (i.e., the *Q*_M_ statistics are not significant), including survival of echinoderms or crustaceans, calcification of corals or mollusks, or growth of corals, echinoderms or mollusks (Fig. 5; Table S4).

**Fig. 6:**
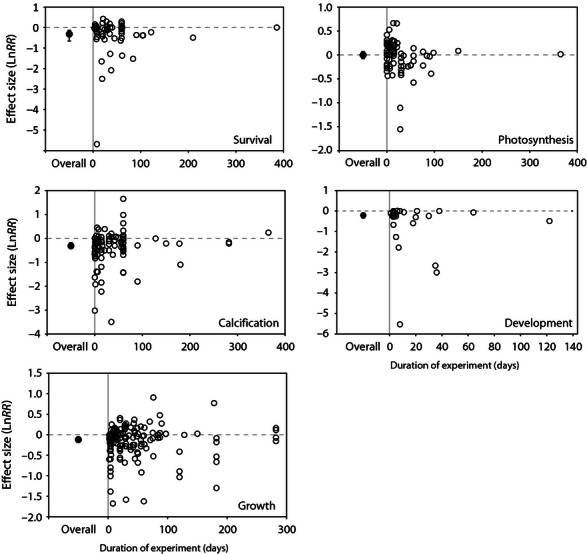
The effect of duration of experimental CO_2_ enrichment on *LnRR*. The mean effect size and 95% CI (for all taxa pooled) is shown on the left of each figure (overall), while the individual LnRR estimates for each study are plotted against duration (days) on the right side of the figure for survival, calcification, growth, photosynthesis and development.

The duration of the experiments are heavily skewed towards shorter experiments (Fig. 6), making inferences regarding the influence of experiment duration on effect size problematic. For most taxonomic groups, significant effects of experiment duration on effect size are not detected, while in some limited cases, there is a small but significant effect (Fig. 6, Table 1). However, the limited number of data points at longer durations strongly influences these patterns, and the shape of the distribution of effect sizes are unknown at longer durations.

**Table 1:** The effect of experiment duration on log-transformed response ratio from continuous random effect weighted meta-analysis

Response	Taxa	df	Slope	*P*-value
Survival	Mollusks	25	0.008	0.130
	Echinoderms	10	0.010	0.475
	Crustaceans	17	−0.003	0.002*
Calcification	Corals	40	0.001	0.007*
	Coccolithophores	11	0.001	0.103
	Mollusks	17	0.004	0.043*
Growth	Corals	17	0.001	0.270
	Mollusks	42	0.001	0.001*
	Echinoderms	34	−0.002	0.889
Photosynthesis	Calcifying algae	10	−0.001	0.641
	Corals	9	0.000	0.047*
	Coccolithophores	14	0.000	0.017*
	Seagrasses	12	0.007	1.000

* indicates P < 0.05.

The influence of the magnitude of the reduction in seawater pH is not consistent across taxonomic groups and response variables. Similar to the duration analyzes, the effect of the magnitude of the pH change is only detected in a limited number of analyzes (Table 2). These effects are very small, differ in the sign of the slope, and are often heavily influenced by a few responses, analogous to statistical outliers (Figs S3–S6).

**Table 2:** The effect of the magnitude of pH reduction on log-transformed response ratio from continuous random effects weighted meta-analysis

Response	Taxa	df	Slope	*P*-value
Survival	Mollusks	25	−1.124	0.813
	Crustaceans	17	−0.4966	0.145
Calcification	Corals	40	−0.7372	0.668
	Coccolithophores	18	−1.9107	0.009*
	Mollusks	18	1.2947	0.014*
Growth	Corals	17	−2.634	0.274
	Coccolithophores	17	0.1564	0.555
	Mollusks	44	0.2605	0.022*
	Echinoderms	34	0.3034	0.006*
Photosynthesis	Calcifying algae	10	−0.4131	0.782
	Corals	10	0.2816	0.125
	Coccolithophores	18	−0.5652	0.139
	Seagrasses	12	0.2254	0.363

* indicates P < 0.05.

**Fig. 7:**
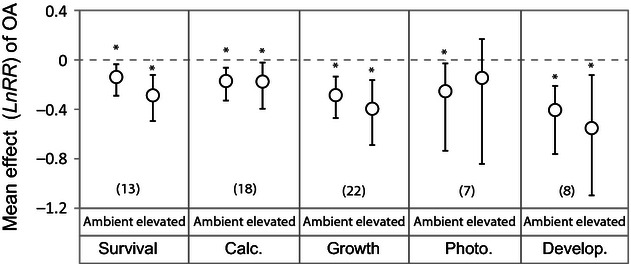
Variation in effect of acidification treatment at ambient temperature and elevated temperature for different response variables**.** Means are from weighted, random effects categorical meta-analyses for each separate response variable. Error bars represent bias-corrected bootstrapped 95% confidence intervals, and the number of experiments used to calculate the means is shown in parentheses. *denotes a significant difference from zero.

There is a trend towards lower survival, growth and development (approximately 8–11%) at elevated temperatures, although these differences are not statistically significant (Fig. 7). Elevated temperature has no clear effect on calcification estimates, and there is a nonstatistically significant trend towards higher photosynthesis in response to acidification in the subset of experiments included in this analysis. However, the differences in effect sizes to exposure to acidification at ambient temperature and at elevated temperature do not explain a significant amount of heterogeneity in any dataset (Table S5).

### Sensitivity analyzes

Rosenthal's fail-safe numbers are large for all analyzes, ranging from 192 to 6157, suggesting that the results are robust. Furthermore, there is no change in significance with the singular removal of any of the experiments with large effect sizes. Therefore, all experiments are included in the analyzes. Additionally, all of the unweighted, fixed effects analyzes reveal very similar patterns to their respective weighted, random effects analyzes (Fig. S1). Finally, while the magnitude of effect size in the duration-weighted survival rate is less than the final estimates of survival, both analyzes reveal very similar patterns (e.g., the significance of the mean effect size did not change for any analysis). The effects of acidification on duration-weighted survival rates are reported in Supporting Information (Fig. S2).

## Conclusion

Our results reveal reductions in survival, calcification, growth, development, and abundance in response to ocean acidification across a broad range of marine organisms. These results support the findings of previous meta-analyzes (Kroeker *et al*., 2010) and suggest that the effect of ocean acidification will be widespread across a diversity of marine life. In addition, the analyzes reveal significant trait-mediated variation in the sensitivity of marine organisms. In general, heavily calcified organisms, including calcified algae, corals, mollusks, and the larval stages of echinoderms, are the most negatively impacted, whereas crustaceans, fish, fleshy algae, seagrasses and diatoms are less affected or even benefit from acidification (Fig. 4) whereas some fleshy algae and diatoms may benefit, although marginally, from the same conditions (Koch *et al*., 2013). These results support previous analyzes despite the tripling of studies and the doubling of species included in the analyzes, suggesting that species' traits (taxonomic group) may be a robust factor for forecasting species sensitivity to acidification.

Most of the mean effect size estimates fall within the 95% confidence intervals of the previous meta-analysis (Kroeker *et al*., 2010), with the exception of crustaceans. The mean effect of acidification on crustacean calcification and growth fall outside of the previous 95% confidence intervals and are more negative in both cases (although not statistically significant) primarily due to the addition of two studies examining barnacles (Findlay *et al*., 2010a,b). These results suggest that the growth and calcification of heavily calcified barnacles may be more susceptible to acidification than other mobile crustaceans. Generally, the other mean effect size estimates (those within previous 95% confidence intervals) do not follow directional patterns (i.e., some increase, whereas others decrease slightly) suggesting that reported patterns are robust.

While the broad scale patterns are robust, new insight is gained by examining the body of studies published in recent years. Whereas the mean effect of acidification on mollusks was not significant for any response variable in a previous meta-analysis (Kroeker *et al*., 2010), the power provided by the additional 39 recent studies published reveal significant reductions in calcification (40%), growth (17%) and development (25%) of this group. When compared with other taxa, these new results suggest that mollusks are one of the groups most sensitive to acidification (Fig. 4), suggesting the exposure of early life stages of mollusks to acidification may represent a bottleneck for their populations (Talmage & Gobler, 2010; Crim *et al*., 2011; Hettinger *et al*., 2012). The slower development of mollusk larvae supports this result as well (Fig. 3). Indeed, the results from the present meta-analysis are consistent with recent evidence suggesting that oyster larvae in hatcheries in the Northeast Pacific Ocean are very sensitive to acidification and are already being impacted by low pH waters (Barton *et al*., 2012). Furthermore, recent studies suggest that carry-over effects between life history stages of mollusks can influence the response at later life stages (Hettinger *et al*., 2012; Parker *et al*., 2012).

The increase in the number of studies considering multi-species responses to acidification allows the first synthetic analysis of abundance patterns. Species abundance patterns are of particular interest, because it integrates many of the physiological effects of acidification, as well as indirect effects via species interactions when quantified in a multi-species assemblage. Most of the abundance estimates in this meta-analysis are from multi-species assemblages (75% for mollusks, 90% for corals and 100% for calcifying algae, crustaceans and fleshy algae), with the exception of coccolithophores and diatoms for which the studies are more often focused on specific growth rates of single species. The results reveal considerably more variability in the effects of acidification on abundance than the other response variables (note the large confidence intervals and larger scale in Fig. 2), especially among mollusks and crustaceans. This suggests that species interactions may decrease the predictability in species responses (Fabricius *et al*., 2011; Hale *et al*., 2011; Kroeker *et al*., 2011b). Indeed, studies examining impacts of acidification on multi-species assemblages have reported opposing responses of closely related species within the same assemblage, potentially due to compensatory dynamics among the most tolerant species (Fabricius *et al*., 2011; Hale *et al*., 2011; Kroeker *et al*., 2011b; Porzio *et al*., 2011). Abundance estimates are based upon results from four field studies in three naturally acidified ecosystems, two field mesocosms, and 29 laboratory studies containing multiple species (Table S1), suggesting the results are not biased by a specific approach.

Another important insight in the abundance analysis concerns the early life stages of corals. All abundance estimates for corals used here are focused on the percent settlement of coral spat (Table S1), whereas other response variables mostly estimate the effects of acidification on adult corals. The effect of acidification on coral abundance was greater than its effect on any other response (e.g., abundance is reduced on average 47%, while other response variables are reduced less than 34%). In several studies, this response was dependent on the exposure of the settlement substrate to reduced pH seawater, suggesting ocean acidification affects coral settlement indirectly by affecting the community composition (primarily crustose coralline algae and/or microbial biofilms) or biological and chemical settlement cues (Albright *et al*., 2010; Albright & Langdon, 2011). These results suggest that the settlement of coral larvae may be particularly sensitive to acidification and could also represent a bottleneck for population dynamics of corals in acidified conditions (Albright *et al*., 2010; Albright & Langdon, 2011; Doropoulos *et al*., 2012).

While the effects of acidification on the early life stages of mollusks and coral settlement (abundance) are significant, the sensitivity of early life stages of other taxa are not clear in other categorical meta-analyzes (Fig. 5). These results suggest that the amount of variation due to differences in sensitivity among life stages may be relatively small compared with other sources of variation for some groups. Thus, it is suggested that the identification of potential life history bottlenecks may be best approached at a finer taxonomic resolution for these groups (i.e., quantifying variation in sensitivity of life stages within specific species).

Although the differences between acidification effects at ambient and elevated temperature do not explain a significant amount of variation, there is a trend towards lower survival, growth and development at elevated temperature. Given the significant variation already attributed to taxonomic groups and life history stages, the inability to detect statistically significant differences does not suggest that increased temperature does not affect the response to ocean acidification. It rather suggests that other sources of variation in these analyzes may be more pronounced than the difference in effect size at ambient and elevated temperatures. However, the trend towards lower survival, growth and development on average at elevated temperatures, suggest that continued research on the combined impacts of acidification and warming may be critical for accurately forecasting marine species responses to acidification in the near future.

When all taxa are pooled together, the effects of elevated temperature on species responses to acidification are clearly not apparent for calcification. Modeling efforts have highlighted how warmer temperatures that increase calcium carbonate precipitation kinetics can potentially offset the reduction in calcification caused by lower pH in some species of corals that are able to up-regulate internal pH (McCulloch *et al*., 2012). However, this response is limited to certain species and to temperature increases that are within the thermal tolerance of the organism (Pörtner, 2008). Nonetheless, the analysis does contain several studies on corals (10 of 18 experiments examined the response of corals), and increased kinetics due to warmer temperatures could in part explain the insensitivity of the acidification-driven calcification response to increased temperature. Additional studies have suggested that temperature and acidification affect different pathways, with temperature overriding the effects on survival (Findlay *et al*., 2010a; Lischka *et al*., 2011) and ocean acidification affecting calcification more specifically. Thus, while there is some evidence for synergistic effects of temperature and acidification in some studies (Reynaud *et al*., 2003; Anthony *et al*., 2008; Rodolfo-Metalpa *et al*., 2010), our results suggest that this is not the norm in experiments examining their combined impact on calcification (see Comeau *et al*., 2010).

While the meta-analyzes can explain some variation in responses based on biological traits, the remaining variation within taxonomic groups is still of real ecological interest. Although this remaining variation could represent species-specific sensitivities, the importance of context has recently become more apparent. For example, the responses of both corals and mussels to acidification have been shown to be dependent on their food supply (Holcomb *et al*., 2010; Melzner *et al*., 2011). Although the available studies are few, we found that the difference in *LnRR* estimates between unfed/low nutrient vs. fed/high nutrient species within in single study can sometimes span or exceed the size of the 95% confidence interval for coral calcification (Holcomb *et al*., 2010; Melzner *et al*., 2011; Edmunds 2011). For example, the range of *LnRR* estimates of coral calcification in zooxanthellate corals (*Astrangia poculata*) between high and low nutrient concentrations (i.e., the difference between high and low nutrient treatments = range = 1.0 *LnRR*; Holcomb *et al*., 2010) is more than double the 95% confidence interval for coral calcification (95% CI = 0.48). However, the range of area-normalized calcification *LnRR* estimates between *Porites* spp. with and without heterotrophic feeding (range = 0.22 *LnRR*; Edmunds 2012) is about half the 95% CI. In another example, the range of growth estimates of mussels (*Mytilus edulis*) between high and low food concentrations (range = 0.08 *LnRR;* Melzner *et al*., 2011) is also approximately half the 95% confidence interval for mollusk growth (95% CI = 0.21 *LnRR*). While the examples are few, these results suggests that nutritional status is not trivial in determining species sensitivity to acidification and should be considered to control for sources of variability.

In addition, populations can be locally adapted to different environmental conditions (Sanford & Kelly, 2010) and respond differently to the same acidification stress (Langer *et al*., 2009; Sunday *et al*., 2011; Pistevos *et al*., 2011; Parker *et al*., 2011). For example, the range of *LnRR* estimates for growth among selectively bred lines of the Sydney rock oyster (range = 0.72 *LnRR*; Parker *et al*., 2011) was over three times the 95% confidence interval for the mean effect of acidification on mollusk growth (95% CI = 0.21 *LnRR*). In another example, the range of *LnRR* estimates for growth of different strains of the coccolithophore *Emiliania huxleyi* (range = 0.47 *LnRR*; Langer *et al*., 2009), almost doubles the 95% confidence interval for coccolithophore growth (95% CI = 0.28 *LnRR*). In many cases, the response of a single population is reported as if it was the response of the entire species. As the field progresses, care must be taken into account for and report factors such as location for source populations and background environmental conditions of source populations (McElhany & Busch, 2012) to refine our understanding of acidification's biological impacts.

Despite the growing interest in acclimation to ocean acidification (Evans & Hofmann, 2012), a signal of acclimation is not clear in this data set (i.e., it is not clear whether organisms exposed to acidification for longer durations are less affected than those in short-term experiments). While the analyzes highlight high variability in the short-term experiments, the few experiments at longer durations fall well within the range of effects in short-term experiments and are still well-estimated by the mean effect sizes (Fig. 6). Additional experiments for extended durations, are needed to understand whether the distribution of effect sizes shifts or becomes smaller (i.e., the variability is reduced) over time. However, field studies have shown that species respond to relatively short fluctuations in carbonate chemistry (e.g., diel fluctuations) even when they experience these conditions regularly (Price *et al*., 2012). Thus, although short-term studies may not address acclimation potential, the results are still informative and can be ecologically relevant.

While the magnitude of the pH change does not consistently explain a significant amount of variability, it does not necessarily indicate that the magnitude of ocean acidification will not influence species responses. Instead, other sources of variation could be masking a potential relationship between the responses of taxonomic groups and the degree of acidification, including methodological sources of error or true biological sources of variation. In addition, the relationship between the magnitude of pH changes and species responses could be nonlinear, and/or more pronounced changes could be detected in lower pH conditions (Scheffer & Carpenter, 2003; Ries *et al*., 2009; Christen *et al*., 2012).

In conclusion, analysis of the rapidly expanding body of research on acidification reveals consistent reductions in calcification, growth, and development of a range of calcified marine organisms, despite the variability in their biology. While our syntheses suggest that some taxa may be predictably more resilient or may benefit from ocean acidification (e.g., brachyuran crustaceans, fish, fleshy algae, and diatoms), it should be noted that a decrease in pH is also likely to have effects that are not captured in the physiological and ecological response variable synthesized here. For example, acidification appears to have neurological effects on fish with repercussions for their behavior (Nilsson *et al*., 2012), whereas some marine plants appear to lose the phenolic compounds used as herbivore deterrents under acidified conditions (Arnold *et al*., 2012). Furthermore, the potential for acclimation (Evans & Hofmann, 2012) or adaptation (Sunday *et al*., 2011; Lohbeck *et al*., 2012) in response to acidification could potentially lessen the effects on calcified taxa synthesized here and remain critical areas for future research. While physiological effects on these calcified organisms can result in decreases in their abundance, the higher variability in species responses in multi-species studies indicates that species interactions will also be important determinants of abundance (Fabricius *et al*., 2011; Kroeker *et al*., 2011b). Furthermore, understanding whether the remaining variation within taxonomic groups and life stages represents real biological differences among species, locally adapted populations, or acclimatory capacities, rather than experimental error, remains a critical area for future research. Finally, marine organisms of the future will not be subjected to acidification in isolation, and our results suggest that continued research on the concurrent effects of warming and acidification is necessary to forecast the status of marine organisms and communities in the near-future.

## Supporting Information

**Figure S1**. Unweighted, fixed effects meta-analyzes.

**Figure S2**. Comparison of total percent survival and calculated daily survival rate estimates (weighted by the duration of the study) pooled for all taxa.

**Figure S3**. Effect of pH change on *LnRR* estimates of survival among taxonomic groups.

**Figure S4**. Effect of pH change on *LnRR* estimates of calcification among taxonomic groups.

**Figure S5**. Effect of pH change on *LnRR* estimates of growth among taxonomic groups.

**Figure S6**. Effect of pH change on *LnRR* estimates of photosynthesis among taxonomic groups.

**Table S1**. Studies used for analyzes (excel file).

**Table S2**. Statistics for overall analyzes.

**Table S3**. Statistics for categorical taxonomic analyzes.

**Table S4**. Statistics for categorical life stage analyzes within taxonomic groups.

**Table S5**. Statistics for categorical temperature analyzes.
