# Supplementary material for: Impacts of ocean acidification on marine organisms: quantifying sensitivities and interaction with warming
Source: Glob Chang Biol. 2013 Apr 3;19(6):1884–96. doi: 10.1111/gcb.12179 (PMC3664023; doi:10.1111/gcb.12179)
Supplement: Supplementary file 1 [file gcb0019-1884-SD1.pdf]

Supplementary Materials
Kroeker et al. Global Change Biology

TABLES

Table S1. Studies used in analyses (see separate excel file)

Table S2. Heterogeneity (Q_T) in overall analyses

Response	Q_T	*df*	*p-value*
Survival	48.38	68	0.966
Calcification	317.92	109	<0.001*
Growth	268.69	172	<0.001*
Photosynthesis	153.89	81	<0.001*
Development	76.40	23	<0.001*
Abundance	198.17	71	<0.001*
Metabolism	27.36	31	0.654

Table S3. Variation explained by taxonomic groups in categorical random effects meta-analysis

Response	Q_M	Q_E	*df*	*p-value*
Calcification	28.70	267.47	5,94	0.065
Growth	70.57	442.64	8,146	0.001*
Photosynthesis	45.85	125.21	8,71	0.005*
Development	9.06	28.04	1,14	0.030*
Abundance	42.55	58.57	6,41	0.020*

Table S4. Variation explained by life stages within taxonomic groups in categorical random effects meta-analysis

Response	Q_M	Q_E	*df*	*p-value*
**Molluscs**				
Survival	0.01	0.0233	2,23	0.018*
Calcification	7.9588	84.9347	1,16	0.297
Growth	3.5581	199.0151	2,41	0.694
Metabolism	15.8214	14.904	1,13	0.003*
**Corals**				
Calcification	0.3	18.25	1,26	0.500
Growth	0.6159	26.2681	1,14	0.560
**Echinoderms**				
Growth	3.2912	105.6343	1,30	0.232

Table S5. Variation explained by temperature treatment in categorical random effects meta-analysis

Response	Q_M	Q_E	*df*	*p-value*
Survival	0.1429	1.3823	1,24	0.124
Calcification	0.0029	52.1375	1,34	0.976
Growth	0.8467	56.3065	1,42	0.424
Photosynthesis	0.3647	28.7812	1,14	0.713
Development	0.6165	21.5299	1,14	0.547

FIGURES

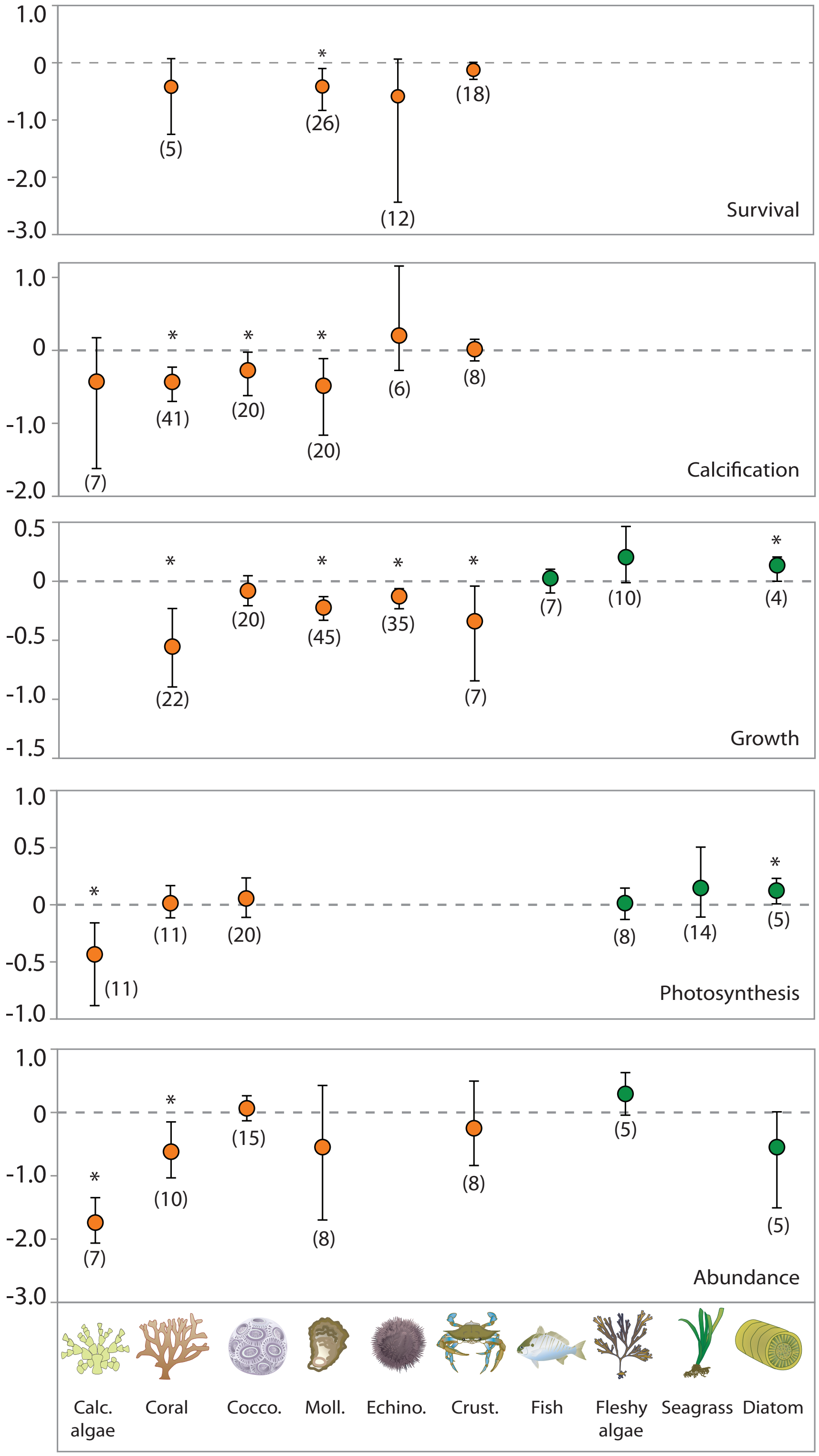

Figure S1. Unweighted, fixed effects meta-analyses of the effects (*LnRR*) of ocean acidification based on broad taxonomic groups.

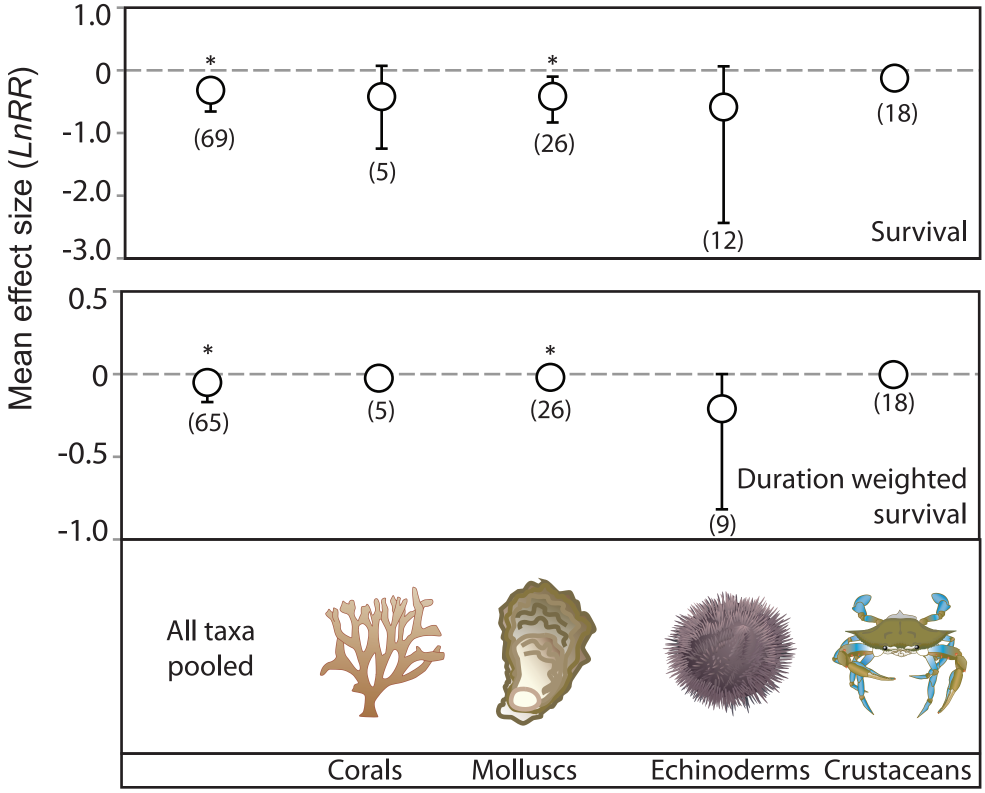

Figure S2. Comparison of total percent survival and calculated daily survival rate estimates (weighted by the duration of the study) pooled for all taxa and key taxonomic groups. *Denotes a significant difference from zero.

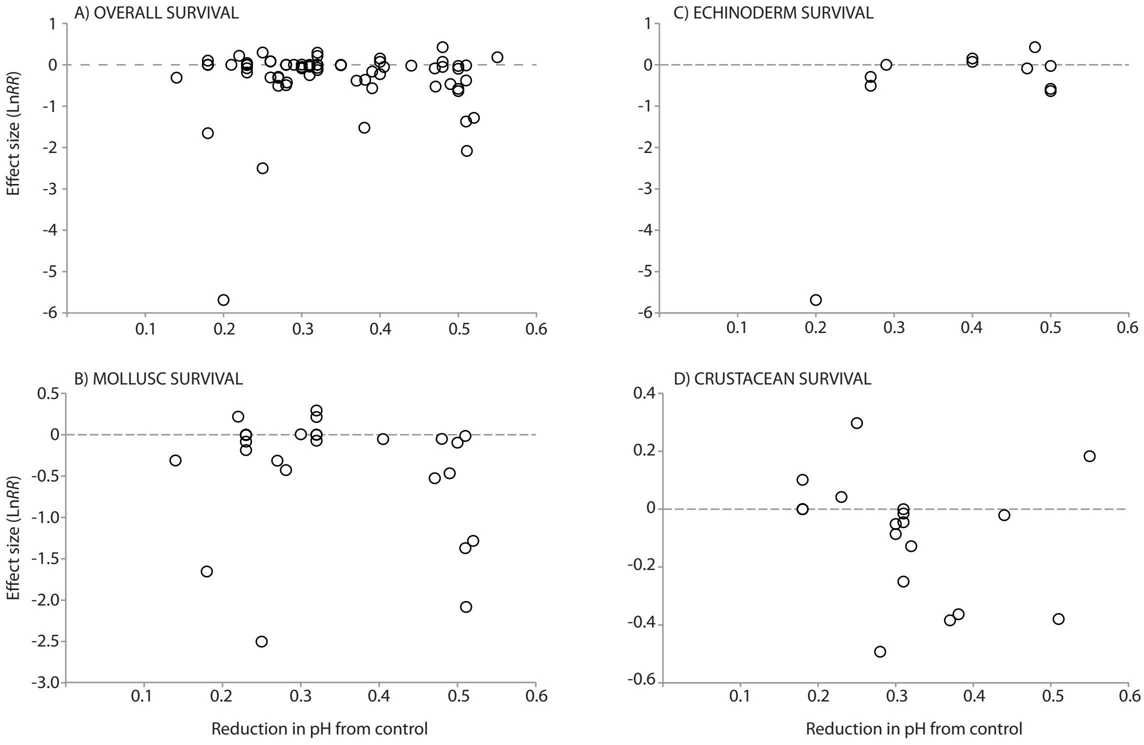

Figure S3. LnRR estimates for survival with individual experiments plotted against the magnitude of the pH change. (A) All taxa pooled together (B) molluscs(C) echinoderms (D) crustaceans.

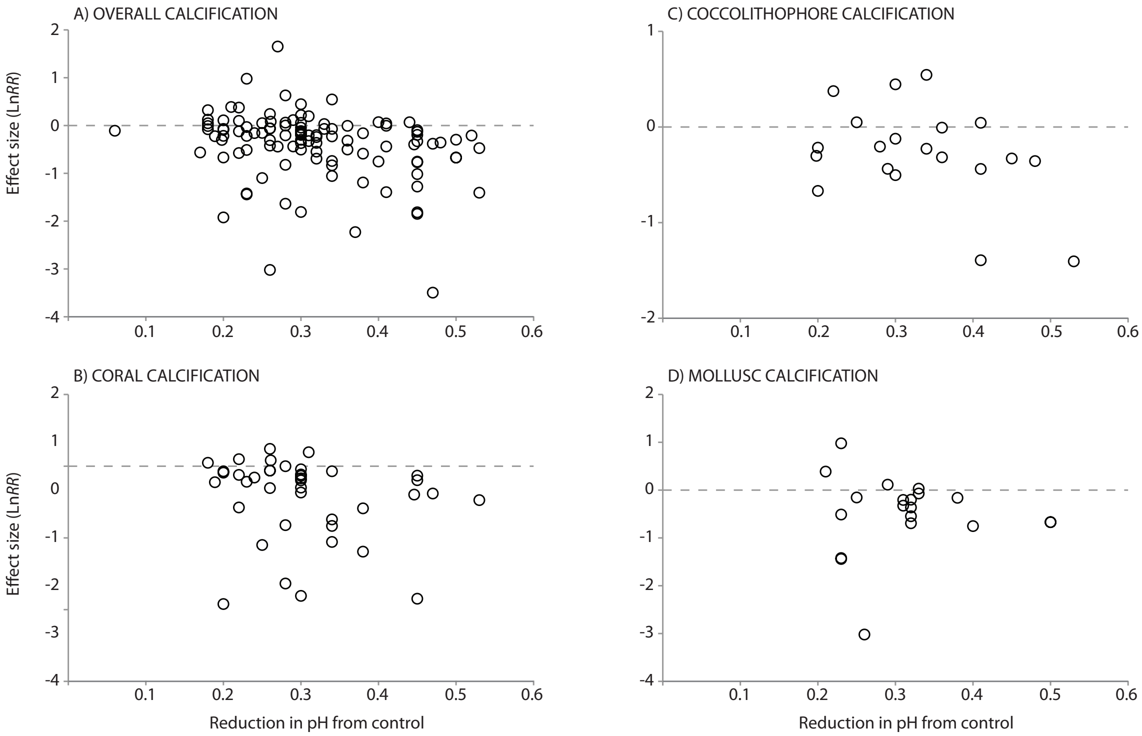

Figure S4. LnRR estimates for calcification with individual experiments plotted against the magnitude of the pH change. (A) All taxa pooled together (B) corals (C) coccolithophores (D) molluscs

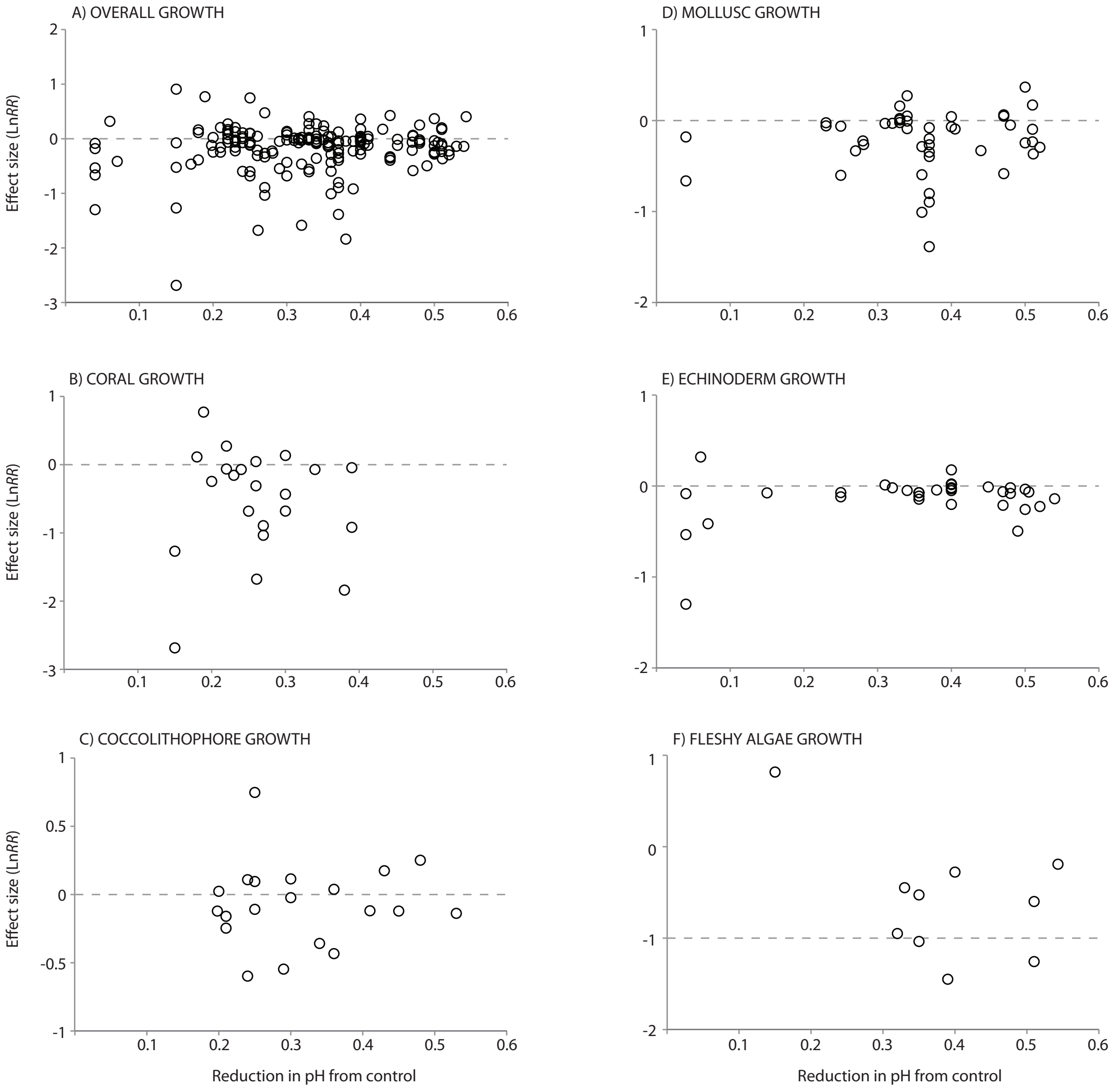

Figure S5. LnRR estimates for growth, with individual experiments plotted against the magnitude of the pH change for growth responses. (A) All taxa pooled together (B) corals (C) coccolithophores (D) molluscs (E) echinoderms (F) fleshy algae.

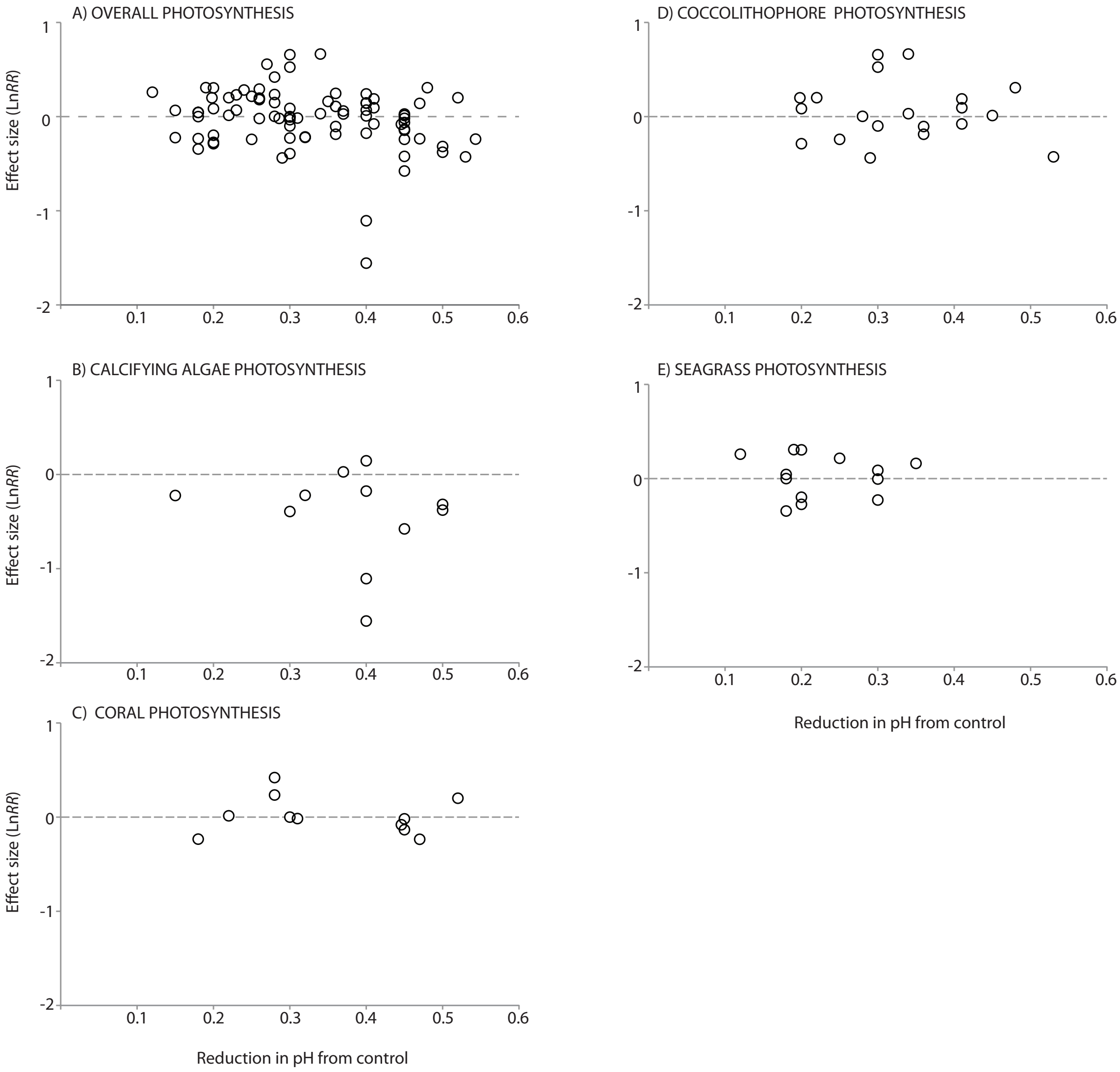

Figure S6. LnRR estimates for photosynthesis, with individual experiments plotted against the magnitude of the pH change for growth responses. (A) All taxa pooled together (B) calcifying algae (C) corals (D) coccolithophores (E) seagrasses.
